# Concerning Boils

**Published:** 1891-02

**Authors:** 


### CONCERNING BOILS.

The predisposing cause of boils is a lowering of the health at some point. The first indication of a boil is a slight itching, followed by a reddish pimple with a hair in the centre. Sometimes the pulling out of this hair will arrest the development of the sore. As the pimple grows, the redness extends and becomes more intense, and the part begins to throb with pain. In about five days it breaks, pus oozes out, the pain abates, and soon after the dead tissue—so called "core"—escapes, followed by rapid healing. Sometimes no core appears, nor does the boil suppurate. This is known as the "blind boil." It is very hard and painful, and is long in healing. A carbuncle somewhat resembles a boil, but is much larger and more painful. It tends to spread, and has several openings. It produces a great disturbance of the whole system, and is very dangerous in its tendency. The constitutional symptoms of boils are slight, though in some cases there may be considerable feverishness. The treatment should be, in the first place, constitutional, aiming to restore the vigor of the system. The diet should be generous, without excess, and easily digestible. The patient, while avoiding exhaustion, should take a proper amount of exercise, have an abundance of pure air, and wash his body daily with cool water and soap. Local treatment also is needed. To arrest the progress of a boil at its early stage, apply a solution of sugar of lead every six or twelve hours with a camel's-hair brush. If this fails, promote suppuration by spreading on it a wash-leather plaster of galbanum and opium, cut in the middle for the escape of the pus. If the pain continues to increase, apply soothing applications When the boil begins to heal, keep the skin around it dry, clean it with tar-soap, and smear it with yellow resin ointment, dressing the broken surface with lint spread with the same ointment, keeping the dressing in position with strips of adhesive plaster or by a light bandage.

